# Gynecomastia: A systematic review of pharmacological treatments

**DOI:** 10.3389/fped.2022.978311

**Published:** 2022-11-01

**Authors:** Ori Berger, Zohar Landau, Ran Talisman

**Affiliations:** ^1^ Plastic Surgery Unit, Barzilai University Hospital Medical Center, Ashkelon, Israel; ^2^ Division of Pediatrics, Barzilai University Medical Center, Ashkelon, Israel; ^3^ Faculty of Health Sciences, Ben-Gurion University of the Negev, Beer-Sheva, Israel

**Keywords:** gynecomastia, pharmacological treatment, adolscents, tamoxifen, selective estrogen receptor modifiers (SERM), aromatase inhibiters, androgens

## Abstract

**Background:**

Pubertal gynecomastia (PG), a benign condition with varied reported prevalence, typically appears at 13–14 years-old and is mostly idiopathic and self-limited. Psychologic impairments are common among adolescents with gynecomastia. Surgical intervention is reserved to severe cases and is offered towards the end of puberty. Pharmacological treatment is seldom given by clinicians mainly due to insufficient published data. We conducted this systematic literature review to assess the efficacy, safety, side effects, and complications of pharmacological treatments published.

**Methods:**

MEDLINE, Embase, and Cochrane CENTRAL were searched for the terms “gynecomastia”, “pubertal”, and “adolescent” in conjunction with medications from the Selective Estrogen Receptor Modulator (SERM), aromatase inhibitors (AI), and androgens groups in different combinations to optimize the search results. Exclusion criteria included: studies based on expert opinion, similar evidence-based medicine levels studies, and studies which discuss gynecomastia in adults. Selected articles were assessed by two authors. Data collected included: the level of evidence, population size, treatment regimen, follow-up, outcomes, complications, and side effects.

**Results:**

Of 1,425 published studies found and examined meticulously by the authors, only 24 publications met all the study research goals. These were divided into 16 publications of patients treated with SERM, of whom four had AI and four androgens. In general, the data regarding pharmacologic therapy for PG is partial, with insufficient evidence-based research. Tamoxifen and SERM drugs have long been used as treatments for PG. Tamoxifen was the chosen drug of treatment in most of the reviewed studies and found to be effective, safe, and with minimal side effects.

**Conclusions:**

Pharmacological treatment as a new standard of care has an advantage in relieving behavioral and psychological distress. Although high quality publications are lacking, pharmacological intervention with tamoxifen is appropriate in select patients. Conduction large-scale high-quality studies are warranted with various drugs.

## Introduction

Gynecomastia, characterized by enlargement of male breast tissue, can be unilateral or bilateral. Male breast enlargement may result from proliferation of ductal or stromal tissue known as true gynecomastia; accumulation of fatty tissue, known as pseudo-gynecomastia; or any combination of these two options (1, 2). Adolescence is one of the three phases in life with the highest incidence of gynecomastia (pubertal or adolescence gynecomastia), with the other two peaks being infancy and old age (2). The reported prevalence of pubertal gynecomastia (PG) is up to 70% (2). The typical onset of true PG occurs at 13–14 years of age, or at Tanner stage 3 or 4, and is followed by a decline of incidence in later teenage years (3). PG develops due to transient imbalances between androgens and estrogens, is idiopathic in over 95% of the patients, and is considered physiological.

Pathological causes of gynecomastia in adolescents are uncommon (less than 5%) and may arise from a broad array of pathological conditions: primary gonadal failure (e.g., Klinefelter syndrome, congenital anorchia), secondary gonadal failure (e.g., insult to the hypothalamic-pituitary axis), tumors (e.g., hCG-producing tumors), defects in androgens synthesis or function (e.g., 11-beta hydroxylase deficiency, androgen insensitivity syndrome, exposure to various drugs (e.g., estrogens, anabolic androgens), and other rare causes (e.g., aromatase excess syndrome) (4).

Gynecomastia is usually asymptomatic; however, it can be associated with pain and tenderness of the mammary gland (5). Psychologic impairments due to a disturbed body image are common and include depression, anxiety, lower self-esteem, identity confusion, eating disorders, social phobia, avoidance, and more (6).

PG is self-limited in 75% to 90% of adolescents and regresses over 1 to 3 years (4). In most patients, careful follow-up and reassurance is sufficient. In severe gynecomastia and/or significant psychological distress surgical or pharmacological treatment should be considered (7–9). There are no exact recommendations for timing of surgical management, but surgery may be considered in adolescents who present with persistent breast enlargement after a period of observation of at least 12 months, intractable breast pain or tenderness, and/or significant psychosocial distress (4).

In the last decades, several studies have examined various pharmacological agents as therapeutic options for treatment of PG. However, solid evidence-based data is minimal and insufficient to establish a standard-of-care (5, 10). In general, medical treatment of gynecomastia aims to correct the estrogen-androgen imbalance and mitigate the effect of postulated estrogen excess by three possible mechanisms: (a) Selective estrogen receptor modulator (SERM), by blocking the effects of estrogens on the breast (e.g., clomiphene, tamoxifen, raloxifene); (b) Androgens (e.g., danazol), by directly increasing androgens concentration and thus rebalancing the androgen-estrogen ratio; and (c) Aromatase inhibitors, acting to inhibit estrogen production by inhibiting aromatization of testosterone and suppressing estrogen production (e.g., anastrozole, testolactone).

Common to all studies regarding pharmaceutical options, is the conclusion that there is a need for additional solid evidence-based studies for the treatment of gynecomastia (5, 8, 11). We conducted this systematic review of literature to assess the efficacy, safety, and side effects of published pharmacological treatment for PG.

## Materials and methods

A comprehensive literature search of MEDLINE, Embase, and Cochrane CENTRAL databases was conducted during December 2021. Search strategies were developed by an information specialist, optimizing the utilization of controlled vocabulary and key words. Electronic inquiries were not limited by publication date or any language restrictions. Drugs of interest included medications from the selective estrogen receptor modulator, aromatase inhibitors, and androgens groups. These terms were searched in conjunction with the terms “pubertal” and “gynecomastia” and their different synonyms (Table 1).

**Table 1:** Database search strategy, search algorithms and terms used to search in the different data bases: (a) Ovid MEDLINE, (b) Embase, and (c) Cochrane CENTRAL.

a. Database: Ovid MEDLINE (R) and Epub Ahead of Print, In-Process, In-Data-Review & Other Non-Indexed Citations and Daily <1946 to December 27, 2021> Search Strategy: 1. (gynecomastia or gynaecomastia or gynecomasty or gynaecomasty).mp. [mp = title, abstract, original title, name of substance word, subject heading word, floating sub-heading word, keyword heading word, organism supplementary concept word, protocol supplementary concept word, rare disease supplementary concept word, unique identifier, synonyms] 2. (antiestrogen* or antioestrogen* or “anti-estrogen*” or “anti-oestrogen*” or (estrogen* adj2 suppress*) or (oestrogen* adj2 suppress*) or tamoxifen or raloxifene or clomifene or clomiphene).mp. 3. (androgen* or danazol or testosterone or androstanolone or dihydrotestosterone).mp. 4. (“aromatase inhibitor*” or letrozole or anastrozole or testolactone or exemestane).mp. 5. 2 or 3 or 4 6. (pubert* or adolescen*).mp. 7. 1 and 5 and 6
b. Database: Embase Search Strategy: #1. Gynecomastia OR gynaecomastia OR gynecomasty OR gynaecomasty #2. Antiestrogen* OR antioestrogen* OR “anti-estrogen*” OR “anti-oestrogen*” OR (estrogen* NEAR/2 suppress*) OR (oestrogen* NEAR/2 suppress*) OR tamoxifen OR raloxifene OR clomifene OR clomiphene #3. Androgen* OR danazol OR testosterone OR androstanolone OR dihydrotestosterone #4. “aromatase inhibitor*” OR letrozole OR anastrozole OR testolactone OR exemestane #5. #2 OR #3 OR #4 #6. Pubert* OR adolescen* #7. #1 AND #5 AND #6
c. Database: Cochrane CENTRAL Search Strategy: 1. gynecomastia OR gynaecomastia OR gynecomasty OR gynaecomasty 2. antiestrogen* OR antioestrogen* OR “anti-estrogen*” OR “anti-oestrogen*” OR (estrogen* NEAR/2 suppress*) OR (oestrogen* NEAR/2 suppress*) OR tamoxifen OR raloxifene OR clomifene OR clomiphene 3. androgen* OR danazol OR testosterone OR androstanolone OR dihydrotestosterone 4. “aromatase inhibitor*” OR letrozole OR anastrozole OR testolactone OR exemestane 5. #2 OR #3 OR #4 6. pubert* OR adolescen* 7. #1 AND #5 AND #6 in Trials

Duplicate records were automatically deleted, followed by manual scanning. The reference lists of the selected publications were screened for additional publications, but none were found. Several selection criteria had to be met for inclusion: studies focusing on PG in adolescents, use of pharmacological therapeutic interventions, and assessments of outcomes. Exclusion criteria included: non-idiopathic gynecomastia, study populations composed of adults only, and reviews that quote previous studies.

Following the primary search, all selected articles were independently assessed by two of the authors using the study criteria. The collected data included: evidence strength, number of patients, treatment regimen, follow-up, outcomes, side effects, and complications. In studies with a mixed age range of patients, information was gathered only from patients under 18 years old. Disagreement on inclusion was resolved by consensus, with involvement of an independent third researcher.

## Results

**Figure 1:**
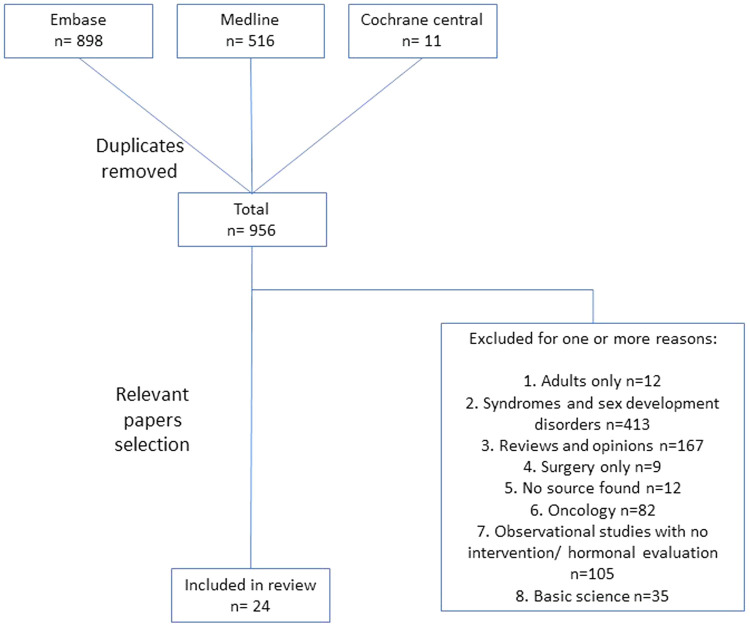
Flow chart of the review process.

Our search methods yielded 1,425 studies; of them 466 were duplicates, leaving 959 studies for review. Of the 24 publications that met the study inclusion criteria, as summarized in Figure 1, 16 examined selective estrogen receptor modulator (SERM), mainly tamoxifen; four aromatase inhibitors (AI), predominantly anastrozole; and the remaining four various androgens (Supplementary Table 1). In the following sections we describe the findings regarding the various aspects of pharmacological treatment of PG according to the different families of drugs.

### Population size and demographics

#### SERM

Tamoxifen was given to 160 adolescent patients, aged 10–19 years (11–22). Raloxifene was given to 25 subjects aged 12–16.6 years (11, 23). Clomiphene was given to 53 patients aged 12–24 years old (24–26).

#### Aromatase inhibitors

Anastrozole was given to 86 patients, aged 11–18 years (27–29). In the testolactone study (30), there were twenty-two patients aged 13.6–23.1 years.

#### Androgens

Dihydrotestosterone (DHT) was given to 13 patients aged 14.8–18 years (31, 32). Patients were given DHT through different methods: intramuscularly every couple of weeks, or with application of daily topical gel (31, 32). Danazol was given to five patients aged 11.7–16.6 years (33), and 11 adolescents (no numerical age specified) (34).

### Breast size change

Different methods were used to measure breast size and changes in breast size: palpation (11, 15–22, 24–26, 29–32, 34), tape or ruler (12–14, 33), ultrasound (28), or a combination of methods (23, 27). Size reduction outcome was defined differently among publications: a noticeable reduction (12–17, 20–22, 24, 25, 29–31, 33, 34), reduction of at least 20% (18, 26); and 50% reduction in breast size (11, 19, 23, 27, 28, 32).

#### SERMs

SERMs had different success rates. Using tamoxifen produced significant changes in 74%–95% of patients (12–17, 19) and a decrease of at least 50% was observed in 41%–77.5% of subjects (11, 22). Raloxifene accounted for at least 50% reduction in 86%–93% of patients (11, 23). Using Clomiphene, a reduction was noted in 64%–95% of cases (24, 25), with a visible change of more than 20% in size in more than 40% of patients (26). Reduction in breast size using SERMs was most often noted after 3–4 months of therapy (11, 14, 15, 23, 25).

#### Aromatase inhibitors

Size reduction in patients treated with anastrozole occurred in 36.1%–72.2% (27–29) of patients, with a good response observed after one month of treatment (29); while with testolactone, a noticeable change was noted in 90% of subjects (29).

#### Androgens

A 72.5%–100% significant reduction rate with DHT (31, 32) was noted after 1 month (32) to 4 months of therapy (31), and a 91%–100% significant reduction rate was noted after treatment with Danazol (33, 34).

### Pain resolution

Pain resolution was reported in several studies (14, 17, 19, 20, 25, 29, 32). In studies reported resolution of pain as an outcome, pain resolution was significant in almost all patients and reported between 1 and 2 weeks (32) to 3 months (19), with most of the studies mentioning pain disappearance after approximately 1 month of treatment (14, 17, 20, 25, 29).

### Follow-up and recurrence

#### SERMs

Tamoxifen had a post treatment follow-up for up to 7 years (11–16, 19, 20). The recurrence rate was as high as 14% (11, 12, 14, 19, 22), occurring in one patient immediately after discontinuation of treatment and in another patient after 22 months post treatment with a good response after a second course of tamoxifen (22). Raloxifene post-treatment follow-up was up to 3 years, with no recurrence reported (11, 23). Clomiphene had a post-treatment follow-up of 3–29 months, and an up to 26% recurrence rate that occurred after 2–9 months post treatment with a short course of clomiphene (24, 25).

#### AI

The AI group (anastrozole and testolactone) had no follow up. The recurrence rate under testolactone was 5% in a patient who experienced a quick response and thus discontinued the therapy early in the period of treatment and monitoring (30).

#### Androgens

DHT post treatments follow up continued for an additional 6–24 months (31, 32). Danazol had no post treatment follow-up (34). No recurrence was noted for either drug (31, 33).

### Side effects, discontinuation, and failure of treatment

Most of the side effects seemed not to be related to the drug exposure (e.g., gastroenteritis and upper respiratory symptoms), while side effects like acne and hot flashes are inherent to the drugs' mechanisms of action.

#### SERM

Two studies reported side effects with tamoxifen: diarrhea (*n* = 1), hot flushes (*n* = 4), and hematuria (*n* = 1) (16, 19); all these patients discontinued therapy. No treatment failure with tamoxifen was reported.

#### Clomiphene

Ten patients discontinued therapy due to dissatisfaction and insufficient response and were referred to surgery.

### Raloxifene

No side effects, discontinuation, or treatment failure were reported.

#### AI

Using anastrozole, two publications reported adverse effects (27, 28). One publication reported gastroenteritis and upper respiratory symptoms in 79% of patients (27), while another described mild to moderate side effects to treatment, similarly to the placebo groups (28). The most common adverse effects under treatment were headache 26%, pharyngitis 19%, rhinitis 14%, acne 12%, and sinusitis 9% (28). With testolactone two patients discontinued therapy after 4 months due to lack of response. One of them and another patient were referred to surgery at their request (30).

#### Androgens

Side effects were noted with danazol, such as acne, muscle cramps, weight gain, fatigue, skin oiliness, nausea, sweating, and edema (34).

## Discussion

PG, although a benign finding, can be a source of great distress and discomfort especially during adolescence. Gender unsuitable physical changes in adolescence have the potential to adversely affect psychological and social development. They can lead to problems in interpersonal relations, social phobia or social isolation, and psychological disorders (e.g., depression, anxiety), and significant deterioration in social and academic processes. A correlation was found between the severity of the gynecomastia and the psychologic distress (2, 6–9, 35–37). Mitigating psychological and social problems are the major reasons for medical intervention in PG.

The incidence of PG is highly variable among different studies and reported to be as high as 70% (2). However, Kumanov et al. (38), reporting on the largest cohort of children (*n* = 6,200), found the prevalence to be under 4%. Such a discrepancy could be explained due to different sizes of breast tissue set as a diagnostic criterion among studies; variance in the size of the study population; or a more random selection of subjects and ethnic differences (38). Nonetheless, such a wide difference in incidence indicates the need for further study (2, 38).

In most patients, PG is transient and resolves spontaneously within 1–3 years (2, 7–9). However, in some patients, gynecomastia will persist beyond 2–3 years, and these cases are often referred to surgical treatment (1, 2, 36).

Currently, the standard of care for patients with gynecomastia during adolescence, who are seeking treatment, is reassurance and explanation of the transient nature of their condition and the pain associated with it. Surgery is usually only considered and executed, if indicated, towards the end of puberty (1, 36). The desired pharmaceutical therapy should be efficient and safe, fast acting, with low rate of side effects or complications. Such medical treatment can significantly shorten to alleviate the period of psychological distress and serve as an inexpensive and effective option (6, 7, 35, 37).

The current data regarding pharmacological treatment for PG is limited with no multi-center double-blind studies. Thus, many clinicians worldwide suggest reassurance and referral to surgery in severe cases. The goal of this systematic review was to assess the existing evidence for the use of medications in treating PG, and to examine if this data can ethically support initiation of pharmacological treatment in selected patients.

No agreement exists regarding prior gynecomastia duration and treatment initiation. Some studies found SERMs treatment to be effective regardless to the duration of prior gynecomastia (11, 21, 24, 25), even if stromal fibrosis has occurred (21). On the other hand, one publication (19) found a better response rate to tamoxifen treatment in patients with gynecomastia duration of less than 2 years as compared to those with gynecomastia of 2 years or more (70% and 56%, respectively). There was also a strong inverse correlation between the duration of the gynecomastia and decrease of breast size with anastrozole treatment (29).

The systematic review uncovered only one randomized controlled study on the use of anastrozole (placebo vs. anastrozole) (28), one cohort study on treatment with DHT (32), and two cohorts use of tamoxifen (13, 19) for the treatment of PG. All other papers reported on case reports or case series (11, 12, 14–18, 20–27, 29–31, 33, 34), while two had a control group (11, 18). The general level of information from the gathered studies that met the inclusion criteria is thus low.

In all the included studies the treatment's results was recorded in a dissimilar and an inconsistent manner. Moreover, the standard staging of gynecomastia severity was not measured or noted, and measuring was conducted by different methods. Obviously, this lack of uniformity in assessing the severity of the gynecomastia, and in defining the outcome and effectiveness of the treatment between the different studies, made assessing the results a real challenge. Pain is another method used to assess the treatment success, but pain was measured in only some studies and was subjective in nature, as it was based on self-reports by the patients and often resolves spontaneously.

Following careful analysis of all the published data, three drugs seem to be the most promising: Tamoxifen, raloxifene, and dihydrotestosterone (Table 2). Tamoxifen is the most studied drug, addressed in half of all included studies (11–22), while data on raloxifene and DHT are more limited and lacking with only two studies for each drug. Raloxifene and DHT were superior to tamoxifen in all measured outcomes (size and pain reduction, side effects profile, and recurrence rate), but tamoxifen has a substantially larger number of patients studied, with a longer post treatment follow-up period. Tamoxifen was found to be safe and effective, with a low rate of side effects and no serious long-term complications.

**Table 2:** Pros and cons of each medication.

Drug (ref)	Advantages	Disadvantages
Tamoxifen (11–22)	– Large body of evidence −12/24 studies – Long follow-up of up to 7 years – Good pain resolution (range 50%–100%) – Good rate of size reduction (range 41%–95%) – Low recurrence rate (range 0%–14%) – Almost no side effects (only two studies found any). – No effects on growth – No effects on gonadal or pituitary hormone levels.	– 8% of all reported patients were referred to surgery post-treatment due to dissatisfaction
Raloxifene (11, 23)	– Relative long follow-up (3 years) – High pain resolution (100%) – Good rate of size reduction Tamoxifen [range 86%–93%)] – No reported recurrence – No reported side effects – No effects on growth – No effects on gonadal or pituitary hormone levels.	– Small body of evidence (2 studies) – Four patients referred to surgery post-treatment due to dissatisfaction
Clomiphene (24–26)	– Relative long follow-up (2.4 years) – No effects on gonadal or pituitary hormone levels.No reported side effects	– Small body of evidence (3 studies) – Relative low pain resolution (86%) – Relatively high reported recurrence (26%) – Five patients referred to surgery post-treatment
Anastrozole (27–29)	– High pain resolution (range 80%–100%)	– Small body of evidence (3 studies) – No follow-up, unknown recurrence rate or post treatment complications – Relatively low size reduction (range 36.1%–60%) – Relatively high rate of side effects (up to 79%)
Testolactone (30)	– No reported side effects – Good size reduction (90%)	– Small body of evidence (one study) – No follow-up – Changes in gonadal or pituitary hormonal levels during therapy and no post-therapy data
DHT (31, 32)	– Relative long follow-up (2 years) – High pain resolution (100%) – Good size reduction (range 72.5%–100%) – Good pain resolution (100%) – No recurrence – No reported side effects	– Small body of evidence (2 studies) – More complex drug delivery route (intramuscular, gel) – Changes in gonadal or pituitary hormonal levels during therapy.
Danazol (33, 34)	– Good size reduction (range 80–91%) – No recurrence – No effect on growth	– Small body of evidence (2 studies) – Relatively short follow-up (6 months) – Unclear pain resolution – Reported side effects (up to 27%) – One patient referred to surgery – Changes in gonadal or pituitary hormonal levels

Although gynecomastia is self-limited in most of the cases, treatment is warranted in the challenging cases when patients have a significant breast mound or suffer significant psychological distress (37). Considering all the above, once the diagnosis of PG is made, treatment with tamoxifen may be favorable (6, 7, 19, 35, 36). Therapy should be attempted. notwithstanding all the foregoing. In case when the treatment is unsuccessful, or the condition reoccurs, surgical treatment remains a viable option.

## Conclusions

Data on pharmacologic therapy for PG is partial and scarce, with insufficient high-quality evidence-based original research. SERMs seem to be effective, with no significant side effects, especially tamoxifen, the most extensively studied drug. Tamoxifen requires a short duration of therapy of up to 6 months. Pharmacological treatment as a new standard-of-care will have a clear advantage in relieving behavioral and psychological distress. Although high quality publications are lacking (e.g., randomized control trails), we believe that pharmacological intervention with tamoxifen is appropriate in select patients. Conduction large-scale high-quality studies are warranted with various drugs.

## Data Availability

The raw data supporting the conclusions of this article will be made available by the authors, without undue reservation.
