# Supplementary material for: Gynecomastia: A systematic review of pharmacological treatments
Source: Front Pediatr. 2022 Nov 1;10:978311. doi: 10.3389/fped.2022.978311 (PMC9663914; doi:10.3389/fped.2022.978311)
Supplement: Supplementary file 1 [file Table1.docx]

| **Publication (ref)** | **Medication** | **Design** | **Population** | **Intervention** | **Control** | **Follow-up** | **Results** | | | | **Remarks** |
| --- | --- | --- | --- | --- | --- | --- | --- | --- | --- | --- | --- |
|  |  |  |  |  |  |  | Pain resolution | Swelling reduction rate | Recurrence rate | Side-effects |  |
| Lawrence et al. (11) | Tamoxifen/ Raloxifene | Retrospective case series | 15 patients Tamoxifen,  10 patients Raloxifene. Ages 12-16.6 yrs. | Tamoxifen: 10-20mg x2 a day, 3-9 months (5.1).  Raloxifene: 60mg x1 a day, 3-9 months | 13 patients | 3 years | - | Manually: Tamoxifen 91%, Raloxifene 86%. | 0% | 0% | 40% unsatisfied and referred to surgery |
| Derman et al. (12) | Tamoxifen | Case series | 10 patients, ages 11.5-14 (mean 12.7) yrs. | 10mg x2 a day, 3-8 months (mean 5.7) | None | 2.5-7 yrs | - | 90% Manually | 0% | 0% |  |
| Derman et al. (13) | Tamoxifen | Retrospective cohort | 13 patients, ages 10-15.8 yrs. | 10-20mg x2 a day, up to 8 months | 8 patients | 4-40 months (mean 21) | - | Manually: Tamoxifen 92%, Control 75% | - | 0% |  |
| Derman et al. (14) | Tamoxifen | Case series | 37 patients, ages 10-16 (mean 13.8) yrs. | 10-20mg x2 a day, 2-8 months | None | 2 years | 100% (in 7 patients) | 95% Manually | 5% | 0% |  |
| Konig et al. (15) | Tamoxifen | Case series | 10 patients, ages 13-18.8 yrs. | 20-40mg x2 a day, 2-12 months (5.5) | None | 2-12 months | - | 80% Manually | - | 0% | 6 previously treated with Danazol |
| Akgül et al. (16) | Tamoxifen | Case series | 29 patients, ages 12.6-17.3 yrs. | 10mg x2 a day, up to 6 months | None | 6 months | - | 90% by Ruler (in 20 patients) | 0% | 10% (rash, hematuria) | 3 patients excluded due to side effects, 6 to an early response |
| Zehetner et al. (17) | Tamoxifen | Case report | 1 patient age 14 yrs. | 20mg x1 a day, 6 months | None | None | 100% | 33% Manually | - | 0% |  |
| Cheon et al. (18) | Tamoxifen | Case series | 10 patients, ages 5-18 (mean 14.6) yrs. | No dosage, 2-4 months | 38 patients | None | - | Mean reduction 1.45+-0.69cm | - | 0% | 7 underwent surgery.  Not stated how measured |
| Devoto et al. (19) | Tamoxifen | Cohort study | 27 patients, ages 12-19 yrs. | 20mg 1x a day, 6 months | None | 1 Year | 100% | 77.5% Manually | 3.70% | 4% diarrhea and hot flushes |  |
| Eversmann et al.(20) | Tamoxifen | Case series | 2 adolescents (14 patients in total) | 20mg x1 a day, 36-60 months | None | 4 months | 50% | 0% | 0% | 0% |  |
| Akgül et al.(21) | Tamoxifen | Case series | 3 patients, ages 16-18 yrs. | 10mg x2 a day, 6 months | None | None | - | 100% Manually | - | - | All unsatisfied, referred for surgery |
| Alagaratnam et al. (22) | Tamoxifen | Case series | 14 patients | No dosage, 1-4 months (2.4) | None | None | - | 86% | 14.3% | 0% | Not stated how measured |
| Gherlan et al. (23) | Raloxifene | Case series | 15 adolescents | 60mg x1 a day, 3-9 months | None | None | 100% | 93% by US | - | 0% |  |
| Stepanas et al. (24) | Clomiphene | Case series | 19 patients, ages 12-24 yrs. | 50mg x1 a day, 1-6 months | None | 3-29 months | - | 95% Manually | 26% | 0% | When response occurred, dose reduced to 50mg every other day |
| LeRoith et al. (25) | Clomiphene | Case series | 22 patients, ages 15-19 yrs. | 100mg x1 a day, 6 months | None | 6 months | 86% | 64% | 0% | 0% | Not stated how measured |
| Plourde et al. (26) | Clomiphene | Case series | 12 patients, ages 12-19 (mean 14.9) yrs. | 50mg x1 a day, 1-3 months | None | None | - | 42%. Manually | - | - | 5 referred for surgery. One patient stated satisfaction |
| Mauras et al. (27) | Anastrozole | Case series | 42 patients, ages 11-18 (mean 13) yrs. | 1mg x1 a day, 6 months | None | None | 80% (in 5 patients) | 36.1% by US, 72.2% Manually | - | 79% | Adverse events: gastroenteritis, upper respiratory symptoms |
| Plourde et al. (28) | Anastrozole | Randomized, double blind control trial | 39 patients aged 11-18 (mean 14.7) yrs. | 1mg x1 a day, 6 months | 35 patients | None | Anastrozole- 91%, Control- 100% | By US: Anastrozole 38.5%, control 31.4% | - | Headache 26%, pharyngitis 19%, rhinitis 14%, acne 12%, sinusitis 9%. | Side effects similar in both groups. One testicular enlargement under Anastrozole |
| Riepe et al. (29) | Anastrozole | Case series | 5 patients, ages 12.7-15.5 (mean 13.9) yrs. | 1mg x1 a day, 6 months | None | None | 100% | 60% Manually | - | 0% |  |
| Zachmann et al. (30) | Testolactone | Case series | 22 patients, ages 13.6-23.1 (mean 15.9) yrs. | 150mg x3 a day, 2-6 months | None | None | - | 90% Manually | 5% | 0% |  |
| Eberle et al. (31) | Dihydrotestosterone | Case series | 4 patients, ages 14.8-16 yrs. | 200-400mg IM, every 2-4 weeks, 16 weeks | None | 6-15 months | - | 100% Manually | 0% | 0% |  |
| Kuhn et al. (32) | Dihydrotestosterone | Prospective cohort | 9 out 40 patients, ages 16-18 yrs. | 125mg gel x2 a day, 4-20 weeks | 22 patients | Two years | 100% | 72.5% Manually | 0% | 0% | Gel applied to skin of breast or abdomen |
| Beck et al. (33) | Danazol | Case series | 5 patients, ages 11.7-16.6 (mean 14.2) yrs. | 200mg x1 a day, 180 days | None | 6 months | - | 80% | 0% | 0% | 20% referred to surgery |
| Buckle et al. (34) | Danazol | Case series | 11 adolescents (out of 42 patients) | 200-300mg x2-3 a day, 4-6 months. Reduced to 200mg daily, 4-6 months more. | None | None | Marked lessening | 91% | - | 27% | Side effects included acne, weight gain, skin oiliness |

Abbreviations: US-ultrasound
